# Computed Tomographic Findings Secondary to Dental Pathologies: Comparison between Rabbits and Guinea Pigs

**DOI:** 10.3390/vetsci10120705

**Published:** 2023-12-14

**Authors:** Daniele Petrini, Caterina Puccinelli, Simonetta Citi, Francesca Del Chicca

**Affiliations:** 1 Department of Veterinary Sciences, University of Pisa, Via Livornese lato monte, San Piero a Grado, 56122 Pisa, Italy; 2 Clinic for Diagnostic Imaging, Vetsuisse Faculty, University of Zurich, Winterthurerstrasse 258c, 8057 Zurich, Switzerland

**Keywords:** computed tomography, rabbit, guinea pig, dental secondary lesions

## Abstract

**Simple Summary:**

Computed tomography (CT) is a diagnostic imaging method frequently used for studying the heads of rabbits and guinea pigs, and dental pathologies are a common reason for requesting head CT examinations in these species. This study aimed to identify and characterize dental-related lesions in both species. All dental abnormalities were associated with secondary alteration of the perialveolar bone in rabbits and guinea pigs. The involvement of mandibular teeth was prevalent in both rabbits (81.2%) and guinea pigs (98%), compared to the maxilla. In the maxilla, increased nasal cavity attenuation (rhinitis) was the most frequent abnormality, observed in 60% of rabbits and 83.3% of guinea pigs; moreover, the exophthalmos was more frequent in rabbits (53.3%) compared to guinea pigs. In the mandible, cavernous space-occupying lesions were predominant in both rabbits (92.3%) and guinea pigs (73.3%). Dental issues often lead to secondary lesions of adjacent anatomical structures in both species, and CT examinations proved valuable for detecting these alterations.

**Abstract:**

(1) Background: dental pathologies are the most frequent reason for requesting a CT scan of the head in rabbits and guinea pigs. The study aimed to review head CT exams of both species to identify and characterize lesions secondary to dental disease. (2) Methods: head CT studies of 48 rabbits and 52 guinea pigs with dental pathologies were reviewed. (3) Results: dental abnormalities of mandibular teeth were the most represented, both in rabbits (81.2%) and guinea pigs (98%). The aggressive bone lesion associated with teeth was the more frequently observed bone lesion in rabbits’ mandible and maxilla; in guinea pigs, the more frequent bone lesions were bulging around the roots of the teeth with focal lysis in the maxilla, and without focal lysis in the mandible. In the maxilla, the increased attenuation of nasal cavities (rhinitis) was the most frequently observed abnormality both in rabbits (60%) and guinea pigs (83.3%); the exophthalmos was more represented in rabbits (53.3%). In the mandible, the cavernous space-occupying lesion was more represented both in rabbits (92.3%) and guinea pigs (73.3%). (4) Conclusions: lesions secondary to dental pathologies were often observed both in rabbits and guinea pigs; CT examination has proven to be valuable in detecting secondary alterations in both species.

## 1. Introduction

Rabbits and guinea pigs have open teeth roots, and this leads to the continuous growth of the teeth [1,2]. Thus, dental diseases and associated complications in rabbits and guinea pigs are the most common disorders encountered in small herbivorous mammals, with high prevalence reported in rabbits (90%) [3] compared to guinea pigs (30.3% and 23.4% in two studies, respectively) [4,5]. These conditions can be life-threatening if untreated because the animals can lose the ability to feed.

The diagnosis of dental diseases is usually based on clinical signs, but diagnostic imaging like oral endoscopy, radiography and computed tomography (CT) of the head plays an important role in the diagnostic process. Examination of the skull and teeth through CT is an alternative that may be better than radiography, particularly if there are multiple findings in various maxillary and/or mandibular quadrants. Moreover, with CT, the anatomical structures can be visualized three-dimensionally, avoiding the superimposition of structures present in radiography. Another advantage of CT is the ability to investigate both soft tissues and dental and bony structures using different viewing windows. Moreover, the CT is more effective for diagnosing soft tissue abnormalities or studying nasal structures [6,7,8,9,10]. For the diagnosis and the detailed characterization of dental pathologies, the use of CT is well documented and is considered the diagnostic modality of choice [10,11,12,13].

Dental diseases have often local effects. Coronal or apical elongation of maxillary cheek teeth may secondarily cause epiphora, dacryocystitis, and other ocular symptoms. Excessive elongation of rostral maxillary apices may also determine an involvement of the nasal cavities, leading to secondary respiratory problems [5,14]. Abscessation is also a possible complication of fractured teeth, coronal elongation, or periapical infection [15,16,17,18,19].

Oral disease in rabbits and guinea pigs is well documented in veterinary medicine as primarily dental pathologies or pathologies of non-dental origin [20]; a recent study [21] documented the incidental findings in the CT exam of the skull in rabbits and guinea pigs, such as otitis (media or externa), rhinitis and soft tissues mineralizations.

The aim of this study was to assess the distribution of the dental pathologies in these two different species and to analyze the lesions of other anatomical structures associated with dental pathologies, based on head CT examinations of rabbits and guinea pigs.

## 2. Materials and Methods

### 2.1. Selection and Description of Subjects

The study was retrospective, multicenter, and had a descriptive design. Rabbits and guinea pigs with a CT study of the head showing dental pathology and with complete medical records (signalment, age, clinical symptoms, indications for the study) were included in the study. All animals were home pets and were admitted at the Vetsuisse Faculty, University of Zurich, Switzerland (Institution 1), or at the Department of Veterinary Sciences, Pisa, Italy (Institution 2) between March 2019 and May 2023. Approval by an institutional animal care and use committee or institutional review board was not required. Informed owner consent was obtained for all the patients before the CT exam was performed.

### 2.2. Data Recording and Analysis

Computed tomography exams of the skull were performed at Institution 1 with a 16-slice CT scanner, Brilliance CT, and, after October 2022, with a 64-slice CT scanner, spiral IQon Spectral (Philips, Zurich, Switzerland), with the following settings: slice thickness 0.8 mm, 120 kVp, 146 and 130 mA, Spiral Pitch Factor 0.288. At Institution 2, CT exams were performed with a 16-slice CT scanner, the helical Revolution ACT (GE Medical System, Bergamo, Italy) with the following settings: slice thickness 1.25 mm, 100 kVp, 120 mA, Spiral Pitch Factor 0.75.

All patients were subjected to general anesthesia and were positioned in sternal recumbency. The anesthetic protocols were adjusted for each patient. A premedication with ketamine and dexmedetomidine administered intramuscularly was performed. Preoxygenation with 100% oxygen for 3–5 min before anesthetic induction was performed. Induction of inhalation anesthesia with 5% isoflurane gas delivered in oxygen was performed through an induction chamber. The guinea pigs were placed on a specific face mask and the rabbits underwent tracheal intubation; both species were attached to a nonrebreathing anesthetic circuit for maintenance with isoflurane delivered in oxygen. Vital signs were monitored using a multiparametric monitor.

For the post-contrast studies, 2 mL/kg of iodinated non-ionic contrast medium was injected (Institution 1: Accupaque 350, 350 mg of I/mL, GE Healthcare, Glattbrugg, Switzerland; Institution 2: Iopamiro, 300 mg/mL Bracco, Milan, Italy) as a bolus with a programmable injector at Institution 1 (Accutron CT-D Medtron Injector, SMD Medial AG, Traegerwilen, Switzerland, injection rate of 2 mL/s) and manually at Institution 2. The late post-contrast phase was scanned between 30 and 60 s post-injection.

The data about species, breed, sex, body weight, and age were recorded from the databases of our veterinary hospitals.

All images were analyzed using an open-source dedicated DICOM viewer (Horosproject.org, Annapolis, MD, USA).

The CT images were reviewed by three observers: a European College of Veterinary Diagnostic Imaging (ECVDI)-certified veterinary radiologist (F.D.C.), an Italian Recognized Specialist in Veterinary Radiology (S.C.), and a veterinary radiologist with P.h.D. in diagnostic imaging (C.P.). Following discussion among the 3 observers, the pathological teeth were identified and classified into 5 different groups: involvement of only incisor teeth (IT); only premolar teeth (PT); only molar teeth (MT); premolar and molar teeth (PMT); incisor, premolar and molar teeth (IPMT). Subsequently, we evaluated the presence of CT findings compatible with secondary lesions to dental pathologies; the following CT abnormalities were recorded and classified based on a previous article by Del Chicca et al., 2023 [21].

Two categories of secondary lesions were considered: 1. involvement of maxillary and/or mandibular bone; 2. involvement of adjacent tissues: soft tissues, nasal cavities, nasopharynx, nasolacrimal ducts, eye, and retrobulbar space. The classification used is reported in Table 1. The aggressive bone lesion associated with tooth (3) described for the maxilla/mandible is characterized by moderate or severe bone lysis of the dental adjacent bone, with or without association with periosteal proliferation.

### 2.3. Statistical Analysis

The statistical analysis was performed by a Ph.D. diagnostic imaging veterinarian with expertise in statistics (CP), using statistical analysis software (GraphPad Prism v. 9.0, GraphPad Software Inc., San Diego, CA, USA). The Shapiro–Wilk test was performed to assess the normality of data. Descriptive statistics were performed. For discrete variables such as gender and breed, the frequency of findings and relative frequencies were calculated. The correlation between the age and the number of the pathological teeth of patients included was evaluated using the Spearman test for both species. The possible differences between the number of pathological teeth of males and females was evaluated with the Mann–Whitney test. Values of *p* < 0.05 were considered statistically significant.

## 3. Results

Forty-eight CT exams of rabbits and fifty-two CT examinations of guinea pigs with dental pathologies were included in the study.

The median body weight of the included rabbits was 1800 g (range 1090–4180 g), and the median age was 65.75 months (range 15–115 months). This study included various rabbit breeds, including 13 (27%) dwarf rabbits, 8 (16.7%) lop rabbits, 2 (4.2%) large breeds, 1 (2%) lionheads, and 24 (50%) unspecified rabbit breeds. A total of 27 out of 48 (56.2%) of the rabbits included were male (4 intact, 23 neutered), and 21/48 (43.7%) were female (8 intact, 13 neutered).

The median body weight of the guinea pigs included was 875 g (range 600–1392 g), and the median age was 35.25 months (range 6–96.5 months). In total, 30 out of 52 (57.7%) of the guinea pigs included were male (3 intact, 27 neutered), while 22/52 (42.3%) were female (19 intact, and 3 neutered).

The indications for the CT studies are reported in Table 2.

No significant correlation was observed between age and number of pathological teeth for both species (*p* = 0.6 for rabbits; *p* = 0.09 for guinea pigs). Moreover, no significant differences were found between the number of pathological teeth in males and females (rabbits *p* = 0.4; guinea pigs *p* = 0.4).

A total of 27 out of 48 (56.2%) rabbits and 25/52 (48.1%) guinea pigs had dental lesions involving the maxilla.

A total of 39 out of 48 (81.2%) rabbits and 51/52 (98.1%) guinea pigs had dental lesions involving the mandible.

All patients with dental abnormalities presented associated secondary bone lesions.

A total of 26 out of 48 (54.2%) rabbits and 20/52 (38.5%) guinea pigs showed lesions of remaining adjacent anatomical structures of the maxilla and/or the mandible secondary to dental disease.

### 3.1. Pathological Teeth and Associated Secondary Maxillary and Mandibular Bone Lesions

#### 3.1.1. Pathological Teeth and Secondary Bone Lesions of the Upper Arcade (Maxilla)

A total of 27 rabbits out of 48 (56.2%) had dental lesions involving the maxilla, of which 10/27 (37%) patients presented bilateral lesions.

A total of 25 guinea pigs out of 52 (48.1%) had dental abnormalities involving the maxilla, of which 7/25 (28%) patients had bilateral lesions.

In Table 3, the number of patients with dental abnormalities involving only the maxilla or both maxilla and mandible is reported.

A total of 23 out of 25 (92%) rabbits had only one category of secondary bone lesions (unilateral or bilateral); on the other hand, 2/25 (8%) rabbits had more than one category of secondary bone lesions (unilateral or bilateral).

Only 23/25 (92%) guinea pigs had only one category of secondary bone lesions (unilateral or bilateral); on the other hand, 2/25 (8%) guinea pigs had more than one category of secondary bone lesions (unilateral or bilateral).

For the maxilla, the secondary bone lesion more frequently observed in rabbits was the aggressive bone lesion associated with teeth (3) in 14/25 (56%) patients, while in guinea pigs the most frequently observed lesion was bulging around the tooth root with focal lysis (2) in 20/25 (80%). The bulging around the tooth root without focal lysis (1) was a less represented alteration in rabbits (6/25; 24%), and the second most frequent alteration in guinea pigs (6/25; 24%). Only 1/25 (4%) guinea pigs presented an aggressive bone lesion (3) associated with molar teeth.

**Figure 1:**
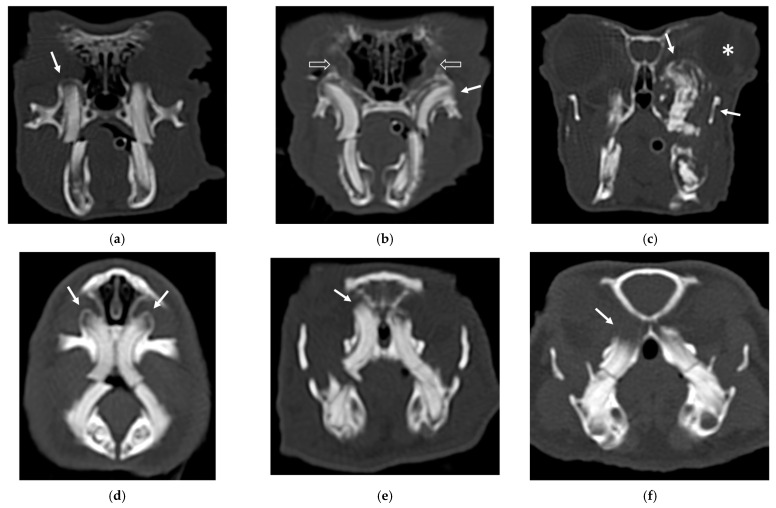
Transverse CT images of maxillary secondary bone lesions of three rabbits (**a**–**c**) and three guinea pigs (**d**–**f**) included in the study; bone algorithm. (**a**,**d**) Bulging around tooth without focal lysis (white arrow); (**b**,**e**) bulging around tooth with focal lysis (white arrow). Note the concomitant presence of nasolacrimalduct enlargement in image (**b**) (empty white arrows); (**c**,**f**) aggressive bone lesion associated with teeth (white arrow). Note the associated exophthalmos in the image (**c**) (white asterisk).

An example of the three categories of secondary bone lesions is shown for rabbits and guinea pigs in Figure 1.

Incisor teeth were pathological in only two cases (one rabbit and one guinea pig) associated with bulging around the tooth root with focal lysis (2). The involvement of the premolar and molar teeth exhibited variable distribution in both species in secondary bone lesions categories 1 and 2.

In Table 4, pathological teeth and associated secondary bone lesions of the maxilla are reported for rabbits and guinea pigs included in the study.

#### 3.1.2. Pathological Teeth and Secondary Bone Lesions of the Lower Arcade (Mandible)

A total of 39 out of 48 (81.2%) rabbits had dental lesions involving the mandible, of which 21/39 (53.8%) patients presented bilateral lesions.

A total of 51 out of 52 (98.1%) guinea pigs had dental abnormalities involving the mandible, of which 41/51 (80.4%) patients had bilateral lesions.

In Table 5, the number of subjects with dental abnormalities involving only the mandible or both maxilla and mandible is reported.

A total of 21 out of 39 (53.8%) rabbits had only one category of secondary bone lesions (unilateral or bilateral); on the other hand, 18/39 (46.1%) rabbits had more than one category of secondary bone lesions (unilateral or bilateral).

A total of 24 out of 51 (47%) guinea pigs presented concomitant dental abnormalities of the maxilla; 27/51 (53%) patients had only involvement of the mandible.

A total of 26 out of 51 (51%) guinea pigs had only one category of secondary bone lesions (unilateral or bilateral); on the other hand, 25/51 (49%) guinea pigs had more than one category of secondary bone lesions (unilateral or bilateral).

For the mandible, the secondary bone lesion more frequently observed in rabbits was the aggressive bone lesion associated with teeth (3) in 24/39 (61.5%) patients. In guinea pigs, the secondary bone lesion more frequently observed was bulging around the tooth root without focal lysis (1) in 37/51 (72.5%).

**Figure 2:**
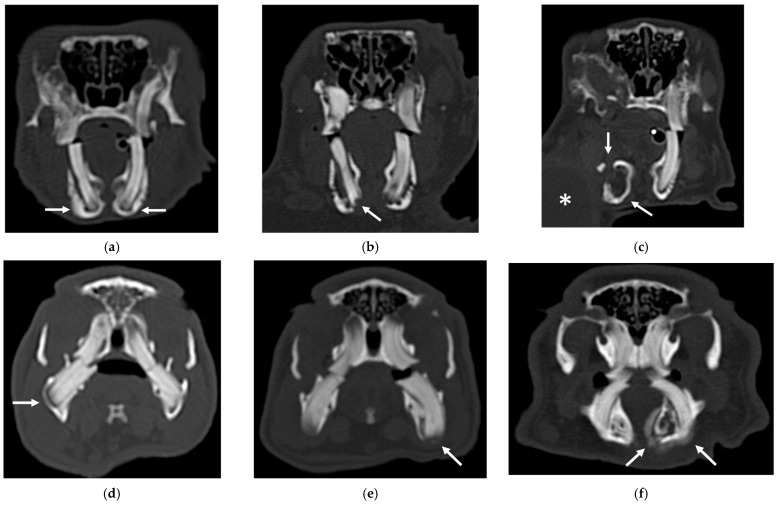
Transverse CT images of mandibular secondary bone lesions of three rabbits (**a**–**c**) and three guinea pigs (**d**–**f**) included in the study; bone algorithm. (**a**,**d**) Bulging around tooth without focal lysis (white arrows); (**b**,**e**) bulging around tooth with focal lysis (white arrow); (**c**,**f**) aggressive bone lesion associated with teeth (white arrow). Note the associated cavernous occupying lesion of soft tissues (white asterisk) in image (**c**).

An example of the three categories of secondary bone lesions is shown for a rabbit and a guinea pig in Figure 2.

The IT were more frequently involved in guinea pigs with aggressive bone lesions associated with teeth (3). In the second group of patients, variable involvement of different groups of teeth was observed. The involvement of the premolar and molar teeth presented variable distribution in both species in all three categories of secondary bone lesions. Only three rabbits and three guinea pigs exhibited involvement of IPMT, associated with aggressive bone lesion associated with teeth (3) in rabbits, and bulging around tooth root with focal lysis (2) and aggressive bone lesion associated with teeth (3) in guinea pigs, respectively, in two and one patients.

In Table 6, pathological teeth and associated secondary bone lesions of the mandible are reported for rabbits and guinea pigs included in the study.

### 3.2. Pathological Teeth and Associated Secondary Lesions of Remaining Adjacent Anatomical Structures

#### 3.2.1. Secondary Lesions of Remaining Adjacent Anatomical Structures of the Upper Arcade (Maxilla)

A total of 13 out of 26 rabbits (50%) had secondary lesions only of the maxillary structures, and 2/26 (7.7%) had secondary lesions of both maxillary and mandibular structures.

A total of 5 out of 20 guinea pigs (25%) had secondary lesions only of the maxillary structures and 1/20 (5%) had secondary bone lesions of both maxillary and mandibular structures.

The pathological teeth and the associated secondary lesions are shown in Table 7.

A total of 15 out of 26 (57.7%) rabbits presented lesions of maxillary structures secondary to dental alterations, of which 7 were right-sided, 5 were left-sided, and 3 were bilateral.

Regarding the nasal cavities, 9/15 (60%) patients had increased attenuation of the nasal cavities (suspected rhinitis) (1), of which 1 was associated with a space-occupying lesion (2) and loss of conchae (3), and 1 was associated with a space-occupying lesion (2). Of the nine patients with suspected rhinitis, six had concomitant pathologies affecting the nasal lacrimal duct, of which one exhibited enlargement (1), one exhibited wall lysis (3), and four exhibited signs of displacement (4).

A total of 8 out of 15 (53.3%) patients had exophthalmos (1), of which 6 rabbits exhibited a concomitant space-occupying lesion in the retrobulbar space (2) and in 1 case, displacement of the nasopharynx (1) was also observed secondary to the space-occupying lesion.

All teeth except incisors were affected; 11 out of 15 (73.3%) patients presented aggressive bone lesions associated with teeth (3) and 4 out of 15 (26.7%) patients exhibited bulging around tooth root with focal lysis (1).

A total of 6 out of 20 (30%) guinea pigs presented secondary bone lesions of maxillary structures; 4 were right-sided, 1 was left-sided, and 1 was bilateral.

A total of 5 out of 6 (83.3%) animals showed increased attenuation in the nasal cavities (suspected rhinitis) (1), of which 1/6 (16.7%) also had exophthalmos (1) associated with space-occupying lesion in the retrobulbar space (2). One patient presented with only exophthalmos (1).

None of the six patients exhibited alteration to the nasolacrimal duct and nasopharynx.

The teeth involved were PT and MT alone or in association. Five out of six patients presented bulging around tooth root with focal lysis (2) and 1/6 patients had aggressive bone lesions associated with their teeth (3).

#### 3.2.2. Secondary Lesions of Remaining Adjacent Anatomical Structures of the Lower Arcade (Mandible)

A total of 11 out of 26 rabbits (42.3%) had secondary lesions of only mandibular structures and 2/26 (7.7%) had secondary bone lesions of both maxillary and mandibular structures.

A total of 14 out of 20 guinea pigs (70%) had secondary lesions of only mandibular structures and only 1/20 (5%) had secondary bone lesions of both maxillary and mandibular structures.

The pathological teeth and the associated secondary lesions are shown in Table 8.

A total of 13 out of 26 (50%) rabbits exhibited secondary bone lesions of the mandible identified as aggressive bone lesion associated with teeth (3) in 12/13 patients and bulging with lysis (2) in 1/13 patients; 8 were right-sided, 3 were left-sided, and 3 were bilateral.

A total of 12 out of 13 (92.3%) rabbits exhibited cavernous space-occupying lesions of soft tissues (3), and 1/13 (7.7%) rabbits exhibited diffuse swelling of the soft tissues (1).

The teeth involved wer: IPMT in five patients, PT in two patients, MT in four patients, and PMT in two patients. A total of 12 out of 13 (92.3%) rabbits had aggressive bone lesions associated with teeth (3).

A total of 15 out of 20 (75%) guinea pigs had aggressive bone lesions associated with teeth (3), of which 7 were left-sided and 8 were right-sided.

All patients also had lesions of the soft tissues: 11/15 (73.3%) patients had a cavernous space-occupying lesion, 1/15 (6.6%) patients had a solid space-occupying lesion and 1/15 (6.6%) animals exhibited diffuse swelling of the soft tissue. The case with the solid space-occupying lesion also exhibited displacement of the hyoid bone.

The teeth involved were IT, PT and MT. All patients had aggressive bone lesions associated with teeth (3).

## 4. Discussion

CT scans of the skull are commonly requested for pets exhibiting symptoms indicative of dental issues, such as epiphora, exophthalmos, changes in the shape of the maxillary or mandibular bones, or the presence of abscesses. Guinea pigs and rabbits are particularly prone to dental problems, and our study aimed to examine and describe the secondary alterations resulting from dental issues that affect the maxillary and mandibular bones, as well as adjacent anatomical structures. Additionally, we aimed to discern any distinctions between the two species in this regard.

In all the patients included in this study, dental abnormalities were consistently accompanied by bone lesions in either the maxilla or the mandible. Among both rabbits and guinea pigs, mandibular abnormalities were more prevalent than maxillary abnormalities.

In rabbits, the most commonly observed secondary bone lesion was the aggressive lesion associated with teeth (3), which affected both the maxilla and the mandible. Conversely, in guinea pigs, the more frequent secondary bone lesions were bulging around the tooth root with focal lysis (2) and bulging around the tooth root without focal lysis (1), affecting the maxilla and mandible, respectively.

It is possible that CT examinations of rabbit skulls were performed at a more advanced stage of dental pathology, potentially due to a delayed onset of symptoms related to dental issues compared to guinea pigs. This assumption suggests that an aggressive bone lesion might be preceded by a less severe bone alteration, such as bulging around the tooth root with or without lysis. It is interesting to note that only the categories bulging around the tooth root with focal lysis (2) and aggressive bone lesion associated with teeth (3) have determined alterations of adjacent anatomic structures. Conversely, the bulging around the tooth root without focal lysis (1) has never been associated with these types of alterations. Performing repeated CT scans of the skull in patients displaying bulging around the tooth root without focal lysis (1) could prove beneficial, particularly if there is a possibility that this condition might progress into a more severe disruption of the perialveolar bone.

The incisor teeth in the upper dental arcade were relatively less affected by dental pathologies and secondary bone lesions in both species. Conversely, the incisor teeth in the lower dental arcade were frequently afflicted in guinea pigs, exhibiting a higher frequency compared to rabbits. While the maxillary and mandibular incisor teeth in rabbits have crowns of equal length, guinea pigs typically possess mandibular incisor crowns that are longer than those in the maxilla [22,23]. The discrepancy in incisor teeth involvement between rabbits and guinea pigs might be attributed to guinea pigs housed in cages, potentially biting the cage bars. In such cases, the presence of longer lower incisors could lead to increased involvement of these teeth.

The secondary involvement of maxillary adjacent anatomical structures exhibited a higher frequency in rabbits compared to guinea pigs, with prevalent lesions represented by exophthalmos and increased nasal cavity attenuation, indicating suspected rhinitis.

Notably, in rabbits, the anatomical positioning of the eyeballs is more dorsally situated in relation to the roots of premolar and molar teeth. In contrast, in guinea pigs, the eyeballs are positioned more laterally. This anatomical difference may account for the elevated frequency of exophthalmos in rabbits, where the alterations of the retrobulbar space could more easily cause eyeball dislocation, as already described in previous literature [24].

The presence of increased nasal cavity attenuation was observed both in rabbits and in guinea pigs. Due to the intimate anatomical proximity of dental structures to the nasal cavities, advanced or severe dental disease can have an indirect impact on nasal structures [24]. It is important to notice that, in these patients, the inflammatory process affecting the nasal cavities can also be primary and not necessarily secondary to dental diseases [21,25].

In 6/15 (40%) of rabbits with abnormalities of maxillary structures, lesions involving the nasolacrimal duct were observed; on the other hand, in guinea pigs, involvement of this structure was not observed in any cases. Anatomically, in rabbits, the incisors and premolars’ roots are in proximity with the anatomical localization of the nasolacrimal duct, predisposing, in case of pathological elongation of the roots, morphological occlusion of the nasolacrimal duct [26,27,28,29,30]. On the other hand, in guinea pigs, no alterations of the nasolacrimal duct related to premolar diseases are described in the literature. In this species, the premolar and molar root teeth are more oblique, and it is possible that in case of elongation of these teeth, the probability of involvement of the nasolacrimal duct is lower compared to rabbits.

The secondary involvement of mandibular adjacent anatomical structures exhibited a higher frequency in guinea pigs compared to rabbits, with prevalent lesions represented by cavernous space-occupying lesions compatible with an abscess in both species. The secondary bone lesion more frequently represented in rabbits and guinea pigs was the aggressive bone lesion associated with teeth (3).

This study has some limitations. First, this is a retrospective study, and this could have had an influence in the selection of the population included in the study. Second, the difference in CT equipment used in the study could be considered a limitation; indeed, different CT protocols could determine a variability in the sensitivity in finding recognition and diagnostic ability. Finally, not all subjects included in the study had a post-contrast CT scan of the head. For the evaluation of only dental and bone disease, contrast studies may not be considered fundamental. The soft tissue abnormalities reported in this study are often recognizable in the pre-contrast study. However, it cannot be excluded that post-contrast studies may have yielded additional useful information.

In conclusion, CT exams of the skulls of rabbits and guinea pigs with dental pathologies have proven to be valuable in detecting secondary alterations in both species. These examinations consistently revealed periodontal bone alterations in all included patients and indicated a higher degree of involvement of adjacent anatomical structures in rabbits compared to guinea pigs.

## Figures and Tables

**Table 1:** Description and classification of the CT findings of the CT studies in rabbits and guinea pigs; in brackets, the identification number of the lesion category.

**Organ**	**Secondary Maxillary and Mandibular Bone Lesions**			
Maxilla/Mandible	Bulging around tooth root without focal lysis (1)	Bulging around tooth root with focal lysis (2)	Aggressive bone lesions associated with teeth (3)	
**Organ**	**Secondary Lesions of Remaining Adjacent Anatomical Structures**			
Nasal cavities	Increased attenuation (rhinitis) (1)	Space-occupying lesion (2)	Loss of conchae (3)	
Nasolacrimal duct	Enlargement (1)	Wall thickening (2)	Wall lysis (3)	Displacement (4)
Nasopharinx	Displacement (1)			
Retrobulbar space	Space-occupying lesion (1)			
Eye	Exophthalmos (1)			
Soft tissues	Diffuse swelling (1)	Solid space-occupying lesion (2)	Cavernous space occupying lesion (3)	

**Table 2:** Indications for the CT studies in rabbits and guinea pigs; “-”: no subject.

	Rabbits (No. of Subjects)	Guinea Pigs (No. of Subjects)
Dental pathology	16/48 (33.3%)	16/52 (30.7%)
Anorexia	6/48 (12.5%)	8/52 (15.4%)
Swelling	12/48 (25%)	20/52 (30.4%)
Nasal discharge	7/48 (14.6%)	2/52 (3.8%)
Exophthalmos	2/48 (2.1%)	2/52 (3.8%)
Ocular discharge	3/48 (6.2%)	-
Weight loss	-	4/52 (7.7%)

**Table 3:** Number of subjects with dental abnormalities involving only maxilla or maxilla and mandible.

Species	Maxilla (No. of Subjects)	Maxilla and Mandible (No. of Subjects)
Rabbits	7/27 (25.9%)	20/27 (74%)
Guinea pigs	1/25 (4%)	24/25 (96%)

**Table 4:** Pathological teeth of the maxilla and associated secondary bone lesions for rabbits and guinea pigs. IT: only incisor teeth; PT: only premolar teeth; MT: only molar teeth; PMT: premolar and molar teeth; IPMT: incisor, premolar and molar teeth; “–”: no subject.

Secondary Bone Lesion	Species	No. of Subjects	Groups of Teeth Involved (No. of Subjects)				
			IT	PT	MT	PMT	IPMT
1	Rabbits	4	–	1	5	–	–
	Guinea pigs	6	–	2	3	3	–
2	Rabbits	10	1	–	7	6	2
	Guinea pigs	20	1	–	13	9	–
3	Rabbits	14	–	7	4	3	–
	Guinea Pigs	1	–	–	1	–	–

**Table 5:** Number of patients with dental abnormalities involving only mandible or maxilla and mandible.

Species	Mandible (No. of Subjects)	Maxilla and Mandible (No. of Subjects)
Rabbits	21/39 (53.8%)	18/39 (46.1%)
Guinea pigs	27/51 (53%)	24/51 (47%)

**Table 6:** Pathological teeth of the mandible and associated secondary bone lesions for rabbits and guinea pigs. IT: only incisor teeth; PT: only premolar teeth; MT: only molar teeth; PMT: premolar and molar teeth; IPMT: incisor, premolar and molar teeth; “–”: no subject.

Secondary Bone Lesion	Species	No. of Subjects	Groups of Teeth Involved (No. of Subjects)				
			IT	PT	MT	PMT	IPMT
1	Rabbits	11	–	4	4	7	–
	Guinea pigs	37	–	33	6	11	–
2	Rabbits	13	1	5	2	8	–
	Guinea pigs	26	1	10	8	11	2
3	Rabbits	24	5	6	8	5	3
	Guinea Pigs	19	12	–	6	1	1

**Table 7:** Secondary lesions of remaining adjacent anatomical structures of the maxilla for rabbits and guinea pigs, and the associated pathological teeth and secondary bone lesions. PT: only premolar teeth; MT: only molar teeth; PMT: premolar and molar teeth; M3: third molar tooth; NDL: nasolacrimal duct. In the columns of the secondary lesions of adjacent anatomical structures, the symbols refer to the number of patients if different to the total number: (α) = 3/4 patients; (β) = 1/4 patients; (χ) = 4/7 patients; (δ) = in 3/7 patients; (ε) = 1/2 patients; “–”: no subject.

Species	Pathological Teeth	Secondary Bone Lesion	Secondary Lesions of Adjacent Anatomical Structures					No. of Subjects
			Nose	NLD	Eye	Retrobulbar Space	Nasopharinx	
Rabbits	PMT	3	–	–	1	–	–	2
	PMT	2	1 (α) 1, 2, 3 (β)	1 (β) 3 (β)	–	–	–	4
	PMT	3	1 (χ)	4 (χ)	1 (δ)	2 (δ)	–	7
	MT	3	1 (ε) 2 (ε)	–	1	2	1 (ε)	2
	MT	3	1		1	2	–	1
Guinea pigs	PMT	2	1	–	–	–	–	4
	MT (M3)	2	–	–	1	–	–	1
	MT (M3)	3	1	–	1	1	–	1

**Table 8:** Secondary remaining adjacent anatomical structures of the mandible for rabbits and guinea pigs, and the associated pathological teeth and secondary bone lesions. PT: only premolar teeth; MT: only molar teeth; PMT: premolar and molar teeth; M3: third molar tooth.

Species	Pathological Teeth	Secondary Bone Lesion	Secondary Lesions of Adjacent Anatomical Structures	No. of Subjects
			Soft Tissues	
Rabbits	PT	3	3	2
	PMT	3	3	2
	MT	3	3	4
	IPMT	2	1	1
	IPMT	3	3	4
Guinea Pigs	IT	3	1	1
	IT	3	2	1
	IT	3	3	6
	PT (P1)	3	3	1
	MT	3	3	6

## Data Availability

The data not presented in the manuscript are available for consultation after a reasonable request to the corresponding author.
